# Brodie’s Lecture on Diseases of the Tongue

**Published:** 1844-08

**Authors:** 


### SELECTIONS

FROM

### AMERICAN AND FOREIGN JOURNALS.

---

*Lectures delivered in the Theatre of St. George's Hospital, in the session of* 1843'–44, by SIR BENJAMIN COLLINS BRODIE, *Consulting Surgeon, of the Hospital.*

*Diseases simulating nasal polypi. Diseased ethmoid bone. Diseases of the tongue. The tongue after salivation and syphilis. Disease simulating cancer of the tongue. True malignant disease of the organ. Removal by ligature. Malignant disease produced by ulcers of the tongue. Ranula. Advantages of a metallic seton over one of silk.*

### DISEASES WHICH ARE SOMETIMES MISTAKEN FOR POLYPI OF THE NOSE.

GENTLEMEN: I have a few words to say concerning these diseases. The case that I shall first mention is a very common one. A young person, frequently a child, is brought, having dilated pupils, a fair complexion, and thin skin, with some difficulty of breathing through the nostrils, and perhaps rather more secretion from them than usual. On looking into the nostrils the Schneiderian membrane appears very turgid, more vascular than ordinary, and on the outside there is a tumor, an excrescence, sometimes small, or at other times pretty large. This may be mistaken for a polypus, and, indeed, the disease puzzled me when I first saw it. This appearance, however, is produced merely by the thickening of the mucous membrene of the nostril at the anterior extremity of the inferior turbinated bone. I do not believe that the mucous membrane there is really more thickened than it is anywhere else; but it is more apparent in that situation on account of the projection of the bone.

In some cases in which the mucous membrane has been sufficiently thickened to obstruct the respiration through the nostril, I have introduced a pair of probe-pointed scissors, slightly curved, and snipped off a portion of the projecting mucous membrane. There is no harm whatever in its excision; and where the nostril is much obstructed, the operation affords great relief. You may suppose this to be a very simple operation; and so it is, for it is done in an instant, but yet it requires some care in order that it may be done properly. In the dead body you might snip off a bit, and if you had not completed it by one incision you could make another. But in the living subject the mucous membrane is full of vessels, and the part must be snipped off at once; for the moment one division is made with the scissors, the hæmorrhage is so great that you cannot see a bit of the remaining part which requires to be divided. It is only every now and then that you find it necessary to have recourse to this operation. In other cases give the child small doses of steel for three weeks, then suspend its exhibition for a fortnight, and again resume it, and proceed in this manner for three or four years. Delicate children who are liable to this disease of the Schneiderian membrane are always benefited by the exhibition of steel; it should, however, be given not in large doses for a short time, but in small ones long continued. Where the constitution is weak, you may sometimes cure the disease in three weeks, but the rectifying of the constitution is a work of years. Some good may be done by local treatment. Dissolve two grains of sulphate of zinc in an ounce of rose-water, and inject a portion into the nostrils two or three times a day; or paint the inside of the nostril with diluted ung. hydrarg. nitratis by means of a camel-hair brush.

I have seen some cases in which a small abscess has formed in the tumor that I have just described. Suppuration has taken place in the substance of the Schneiderian membrane just where it projects in front of the inferior turbinated bone, and the best plan to adopt is to cut off, with a pair of scissors, membrane and abscess together. When an abscess forms in a pile, that is best relieved, not by laying open the abscess, but by snipping off the pile.

Another disease, sometimes mistaken for polypus of the nose, is connected with a morbid condition of the ethmoid bone. A patient has difficulty of breathing through the nose, with pain in the forehead, and blows his nose oftener than natural; by and by he blows away hard dry scabs of mucus, like bits of glue, and then there is an offensive putrid smell perceptible to others, and perhaps to himself also. This indicates disease in the bones of the nose, generally of the ethmoid cells, but sometimes more extensive. Occasionally it is supposed to supervene on syphilis, sometimes it arises from the long-continued use of mercury; sometimes from a scrofulous state of the system; or it may be the result of general bad habit, such as is called *cachexia*. I am not about to enter into a history of this disease at present, but merely to point out that it may be mistaken for polypus. The symptoms always show that it is not a polypus, and if you turn the patient to the light you will see a tumor at the upper part of the nostril. This consists merely of unhealthy granulations, organized lymph covering the Schneiderian membrane over the bone; and if you take hold of it with the forceps you do not pull away a polypus, but a bit of the Schneiderian membrane with the tumor over it. Take care not to mistake this case for polypus, because the treatment that is applicable to the one is quite inapplicable to the other.

Having finished this subject, I propose to call your attention to

*Diseases of the Tongue.*—I am inclined to direct your attention to this subject, first, because you will find, especially in private practice, that you are frequently consulted about diseases of this organ; and, secondly, there is nothing worthy of notice about it in books. I have at different times looked over various books on surgery, and over the journals and surgical reviews, but I have not been able to gain any information about diseases of the tongue, except those of a malignant character; and I must add that the history given, even of these, odd as it may seem, is very different from what I have met with in practice.

In dyspeptic persons the tongue is frequently rather swollen; it becomes cracked on the surface, and may remain so without harm for years. It may bear the appearance of fissures on the surface, and the papillæ may be enlarged. This dyspeptic tongue, existing in a slight degree, is very common. A similar appearance of this organ occurs in persons who have been much under the influence of mercury. When a patient is salivated, the gums become inflamed, and the tongue also becomes inflamed and swollen. In bad cases of salivation, such as you scarcely ever see in the present day, because mercury is more prudently exhibited than formerly, the tongue becomes so swollen that the mouth will not contain it, and this inflammatory state of the organ, arising from the use of mercury, is very apt more or less to persist afterwards. The tongue is swollen; there are fissures on the surface, and this appearance is retained to the last. Sometimes you will see a longitudinal fissure in the median line of the tongue which does not seem to swell up like the rest of the organ. I remember a patient who had thus suffered from the use of mercury; and for a long time afterwards his tongue was much enlarged. The longitudinal fissure was so deep that it looked as if the tongue were divided into two parts, and the patient consulted a medical practitioner, who, not being acquainted with the disease, thought the tongue was going to drop into two pieces, and proposed to fasten it together by a ligature.

This morbid condition of the tongue requires no special treatment. If the patient be dyspeptic, try to put his digestion in as good a state as possible. When he suffers from the use of mercury, give him sarsaparilla, nitric acid, or whatever else may get rid of the effects of mercury.

There are ulcers of the tongue which are different from those I have just mentioned; sometimes they accompany an enlarged and fissured tongue, but they may exist independently of those circumstances. The ulcers to which I now allude, more especially, occur as one of the sequelæ of syphilis. They are sometimes accompanied by the eruptions, little spots of syphilitic psoriasis on the body, and little spots on the scalp, but frequently they occur without symptoms elsewhere. A gentleman whom I saw not long since had a chancre in the spring of the year, some few years ago. Two or three months after that, if I remember aright, he had secondary symptoms. He took mercury, but inadequately; and many months afterwards the secondary symptoms returned; they were, however, but slight, and yielded to some simple treatment. But a year and a half after the first attack of syphilis, there were ulcers of the tongue, so that he could hardly speak or swallow his food; and at the same time spots appeared on his head and elsewhere. He took a little mercury, his tongue got well, and the eruption disappeared. From that time, however, he was subject continually to little ulcers of the tongue, coming but not going of themselves, never disappearing till he had taken blue pill. The ulcers, of which there were several, were very troublesome, interfering with deglutition, nay even making him speak thick, and occasioning him great distress. Of his own accord he took a little mercury when they appeared, and they went away; but in two or three months they were sure to return. At last I made him take a course of grey powder (hyd. c. creta.) for nearly two months; the ulcers healed, and never troubled him again. These cases are very common as a sequel of syphilis, and the ulcers are seldom cured, except by mercury; but, according to my experience, large doses of it do harm rather than good. Calomel and opium is the great medicine to be brought into play in these cases. The mercury with chalk, five grains, with one or two grains of Dover's powder (to prevent it from griping and purging), is preferable to any larger doses of the remedy. The length of time during which a person may be plagued with ulcers of the tongue is astonishing. I have seen them last for years, until a patient has been put through a pretty long course of small doses of mercury. I saw one gentleman in whom these ulcers followed syphilis, and had been going on for two or three years when he came to me. They yielded to the grey powder, but not very rapidly, and the tongue always continued disfigured and covered with cicatrices. In some cases the patient is relieved by sarsaparilla, especially an infusion in lime water. Where mercury has failed I have found that the best remedy is iodide of potassium, two or three grains given twice daily, dissolved in plenty of water; but, in three cases out of four, the grey powder is much more efficacious.

Ulcers of the tongue, such as I have described, sometimes occur merely as accompaniments of dyspepsia, and they generally heal of themselves; but if they do not, one application ot the nitrate of silver is generally sufficient to remove them. Those, however, that follow syphilis, do not yield to this remedy; nor does any local application, so far as I have seen, do much service.

Some persons who have ulcers on the tongue have them also on the inside of the cheek. I suspect that they are originally little eruptions, but as they occur in a mucous membrane they ulcerate more rapidly than they would if they occurred on the skin.

There is a disease of the tongue which I have seen every now and then, and which I am sure is very often mistaken for cancer, though it is of a different nature. It is a curable disease, although it looks like a malignant one in many respects. The first thing of which the patient complains is enlargement of the tongue, with some pain. On examination you find a tumor in one part of it, not very well defined, not with any distinct margin. It is a softish tumor, and increases in size; and perhaps another tumor appears in a different part of the tongue, and that increases also. There may be three or four of these soft elastic tumors, with no very defined margins, in various parts of the tongue. This is the first stage of the disease.

In the second stage there is a small formation of matter in one of these tumors—a little abscess, which breaks externally, discharging two or three drops of pus. When the abscess has burst it does not heal, but another forms in one of the other tumors. These abscesses may assume the form of ulcers, and the ulcer has a particular appearance. In the first instance it is a very narrow streak of ulceration, but on introducing a probe you find that the ulcer is the external orifice to a sort of fissure in the tongue. The probe passes in obliquely; the tongue is, as it were, undermined by the ulcer, a flap of the substance of the tongue being over it.

The disease now becomes more painful, and at last these ulcers may spread externally. In some instances they occupy a very considerable portion of the surface of the tongue, but generally they burrow internally, and do not spread much towards the surface. This is a very distressing state of things, and a man may remain in this state for a long time. The glands of the neck do not become affected, nor does the general health suffer, except from the difficulty of swallowing food. This is one inconvenience experienced by the patient, and he also labors under a difficulty of articulation. The tongue, from its enlarged state, may become stiff, not sufficiently pliable for the purposes of speech, and the patient either speaks thick or lisps.

In some instances the disease may be relieved by a course of sarsaparilla, with small doses of bichloride of mercury. A strong decoction of sarsaparilla, with from a quarter to half a grain of bichloride of mercury, may be taken in the course of the day. Of course, if there be anything wrong in the general health, you should endeavor to get that corrected, and attend especially to the state of the bowels and the secretion of the liver. If the secretions of the digestive organs be unhealthy, a dose of senna and salts may be given every other morning, aud blue pill every other night. When the patient is brought into this state, one remedy, as I have said, is sarsaparilla with bichloride of mercury, but, according to my experience, this is not the best remedy. The remedy best adapted for these cases is a solution of arsenic. Give the patient five minims three times daily, in a draught, gradually increasing the dose to ten minims. It should be taken in full doses, so that it may begin to produce some of its poisonous effects upon the system. When it begins to act as a poison it will show itself in various ways. Sometimes there is a sense of heat, a burning pain in the rectum; sometimes griping, purging, and sickness, and nervous tremblings. A patient who is taking arsenic, especially in pretty large doses, ought to be very carefully watched. At first you may see him every two or three days, and then every day; and as soon as the arsenic begins to operate as a poison, leave it off. When this effect is produced, the disease of the tongue generally gets well, but at any rate leave off the arsenic, and the poisoning will not go too far; it will do no harm. If, after a time, you find that the disease is relieved, but not entirely cured, you may try another course of arsenic. Perhaps it may take a considerable time to get the tongue quite well. Sarsaparilla, with the bichloride of mercury, may be given at one time; and at another, arsenic. You cannot give either of these remedies forever, and indeed the arsenic can only be given for a very limited period; but it is astonishing what bad tongues of this description I have seen get well under these modes of treatment, especially under the use of arsenic.

Malignant diseases of the tongue generally are of the nature of carcinoma, but sometimes of fungus hæmatodes.

Carcinoma generally begins with scirrhous tubercles in the tongue, which may be felt externally; but, from the dissections I have made, I suspect that the disease never begins in one part only; that while there is one tubercle that can be felt, there are others that cannot in various parts of the organ. The scirrhous tubercle increases, becomes attached to the skin, and ulcerates. It may commence in part of the tongue; sometimes the upper part, sometimes the end, and sometimes the lower surface.

Such is the history of the disease as it is commonly given in books, and as it frequently occurs in practice; but I must say that it does not always begin in this manner; and that in many cases a disease which you do not think of any consequence turns out to be malignant. For example; a gentleman came to me with a little round ulcer, not so large as a silver penny, and it gave him no pain. I touched it with the nitrate of silver, and used some other remedies, which I now forget, for it was many years ago. I proposed to remove a part of the tongue by ligature, but he did not like to undergo the operation, and went into the country. I saw nothing of him for three-quarters of a year, and he then came back with an immense ulcer of the tongue. The tongue was much enlarged, and also the glands of the neck. He died, and I made a post-mortem examination. I found an enormous tumor, fungus hæmatodes of the tongue, extending to the epiglottis and the glands of the neck. The only external manifestation of this, in the first instance, was a little ulcer, without surrounding hardnesss, and which yielded to the touch. I

have seen fungus hæmatodes of the glans penis begin in the same manner; it would not heal, and by and by the tumor burst out. A gentleman consulted me about two years ago with some little excrescences on the side of the tongue, which looked so very like warts, that I thought they were so; and I apprehended the disease was malignant, especially as it appeared to be confined to the surface. I am always suspicious of diseases of the tongue. I applied some caustic potassa to the warts, which destroyed them very effectually, and made a deep ulcer there. The part healed, and the patient seemed to be very well. He came to me sometime afterwards with ulcers where the warts had been; there was a great deal of hardness at the base, and they had all the characteristics of carcinomatous ulcers. So they proved to be; the disease continued to spread, there was repeated hæmorrhage, and the patient died. In other cases I have seen disease of the tongue, which did not present a suspicious character at first, prove to be malignant in the end.

There are on the table specimens of malignant disease of the tongue illustrating the progress which I am now going to describe. The ulcer extends, eats away a good bit of the tongue, generally on one side; the organ becomes stiff, gets fixed to the neighboring parts; deglutition and articulation become difficult; the patient complains of pain, and you cannot help him. The ulceration goes on; the constitution suffers from the influence of malignant disease, and also from the want of nourishment; the glands in the neck become affected; and I do not know anything more miserable than a patient dying of malignant ulcer of the tongue. Having described the progress of the disease so far, you can easily conceive the rest. The patient is gradually rendered weaker and weaker, thinner and thinner; then there is a great bleeding; the lingual arteries are ulcerated, and it may be that the patient dies of hæmerrhage, for you can do nothing to stop it except by the actual cautery, and that you are often not in time to apply. The repeated hæmorrhages in these cases generally go a great way towards the destruction of the patient.

In the advanced stage of the disease nothing can be done. Can anything be done in the early stage? Can you remove the scirrhous disease in any way? If it be situated at the anterior part of the tongue you may excise it. An assistant could hold the tongue with a rough towel on one side while you excise the other, and he could also hold it while you secured the bleeding vessels by ligature. But a much simpler way would be to remove the part by ligature. A strong ligature, with a double needle, may be passed through the tongue, and it may include as much as you please. If there be a large portion to be removed, make a notch with a pair of scissors behind and before; into which the ligature can drop so as to enable you to effect the strangulation more completely. It gives a great deal of pain at the time you apply the ligature, but you must have a very strong ligature, and tie it as tight as possible. The part introduced between the ligature is immediately killed; it assumes a purple and then an ash color, and in the course of a few hours the pain is over; but profuse salivation follows, and in some cases lasts two or three days.

There is no difficulty in removing, either by the knife or by ligature, any tumor from the tongue, except it be situated just at the back; but then I must tell you that I never saw any permanent good arise from it in any one instance. In the examinations I have made where there was carcinoma of the tongue, the scirrhous disease was beginning in other parts. A woman has a scirrhous tumor of the breast—do you think that you would succeed in curing the disease by cutting away a portion of the breast and leaving the rest? You have no chance of the operation succeeding, except you remove the whole, unless the scirrhous tumor be distinct with a cyst around it, and have no connection with the breast. If there be fungus hæmatodes of the tibia, no surgeon of sense would think of performing amputation, except above the knee, even if he did it there. In order that an operation for malignant disease may be successful, you must remove the whole of the organ in which it is situated, otherwise there is no chance of permanent good. In the case of malignant disease of the tongue, you cannot remove the whole, but only that little bit in which it has shown itself, while there is an under-current of disease going on everywhere else. I therefore cannot recommend you to perform the operation, and I think it is better to let a disease like this take its course, than to subject the patient to the pain of an operation, and, what is worse, to the disappointment. The patient goes through the operation, and then, in a little while, he is disappointed to find that he is just as bad as ever.

I cannot say that those small ulcers of the tongue which I described before, never run into malignant disease. I suspect that any ulcer there, that has existed for an indefinite time, may assume the character of malignant disease. A patient had ulcers of the tongue and cheek; he was apparently dyspeptic, and, so far as I know, they were not connected with syphilis. He had been subject to them for years, and they generally yielded to some remedies; but at last I was called in to see one of the ulcers unusually intractable in the cheek. It had become malignant, and the patient died of carcinoma of the cheek. Where there are ulcers of the tongue, take care that there are no external causes of irritation acting upon them to keep them up; for this will sometimes convert a simple into a malignant ulcer. Teeth, scarifying ulcers in the tongue, should be extracted. In many cases, rough, ragged teeth, produce disease of the tongue. In malignant disease, I have over and over again, had the teeth extracted, while the event has proved that they might as well have remained; but still, when there is a sharp tooth cutting against the edge of the tongue, you are always to look at it with great suspicion.

There is one other disease of the tongue, or rather a disease under it, which remains to be mentioned. A patient comes with a sore mouth, and you see the tongue pushed up to the soft palate. It looks as if the tongue were enlarged, but that is not the case, it is lifted up. You tell the patient to put his tongue against the incisor teeth, and on looking beneath, you see a tumor. By feeling it, you find fluctuation; you puncture it, and let out a quantity of transparent fluid, sometimes a teaspoonful or more. The fluid is a little glutinous, and consists of saliva. There has been an obstruction to the orifice of the submaxillary gland; the saliva has been secreted by the gland, but could not get out by the duct, and hence it has remained till it has formed a large tumor. This is what is called ranula.

You puncture the tumor with a lancet; the fluid comes out, and immediately the patient is well. You see him a week afterwards; he is quite well, and there is the saliva flowing out of the orifice you have made with the lancet. But you see him a month afterwards, and the tumor has re appeared, the orifice has healed, and the tumor becomes as large as ever. All you want is, to get a permenent orifice from the bag into which the duct has been converted; but that is a very difficult matter. I have tried to effect it in various ways. I have punctured the bag, and then touched the edge with caustic potassa, to prevent its healing. The patient has gone on very well, so long as it did not heal, but as soon as I have left off applying the caustic, the orifice has closed. I have introduced a tenaculum into the bag of the ranula, and cut away a piece sufficiently large to admit the finger; the patient has then continued well for a longer time, because the part takes longer to heal, but contraction takes place, and the patient is bad again. I have run a seton through, and the patient has then gone on well for a considerable time. I have introduced a gold or silver ring, and kept that in as a seton. If the seton be kept in a considerable time, it seems to effect a permanent cure, but even that fails, and you have to perform the operation two or three times. I know of nothing better than the use of a seton, and I believe that it is better made of metallic substance, than of silk. It does not so soon ulcerate its way out, and if it remain in for a long time, the edges of the orifice through which the seton is introduced, may become covered with mucous membrane. If you introduce a silk or India rubber seton in the back of the neck, after a great length of time a sort of skin forms on the inner surface of the canal; there is a discharge of matter; and when you take away the seton, the part in which it lay remains pervious. So if you keep a seton in a ranula, for a very long time, the opening may remain pervious. The advantage of a metallic over a silk seton, is, that it does not ulcerate its way out so soon, does not get putrid in the mouth, and, therefore, may be kept in for a longer time.—*London Lancet.*

